# Multiple Crimean-Congo Hemorrhagic Fever Virus Strains Are Associated with Disease Outbreaks in Sudan, 2008–2009

**DOI:** 10.1371/journal.pntd.0001159

**Published:** 2011-05-31

**Authors:** Imadeldin E. Aradaib, Bobbie R. Erickson, Mubarak S. Karsany, Marina L. Khristova, Rehab M. Elageb, Mohamed E. H. Mohamed, Stuart T. Nichol

**Affiliations:** 1 Molecular Biology Laboratory, Department of Clinical Medicine, Faculty of Veterinary Medicine, University of Khartoum, Khartoum North, Sudan; 2 Molecular Biology Laboratory, Viral Special Pathogens Branch, Division of High Consequence Pathogens and Pathology, United States Centers for Disease Control and Prevention, Atlanta, Georgia, United States of America; 3 Division of Virology, National Medical Health Laboratory, Federal Ministry of Health, Khartoum, Republic of Sudan; 4 Biotechnology Core Facility Branch, Division of Scientific Resources, United States Centers for Disease Control and Prevention, Atlanta, Georgia, United States of America; Tulane School of Public Health and Tropical Medicine, United States of America

## Abstract

**Background:**

Crimean-Congo hemorrhagic fever (CCHF) activity has recently been detected in the Kordufan region of Sudan. Since 2008, several sporadic cases and nosocomial outbreaks associated with high case-fatality have been reported in villages and rural hospitals in the region.

**Principal Findings:**

In the present study, we describe a cluster of cases occurring in June 2009 in Dunkop village, Abyei District, South Kordufan, Sudan. Seven CCHF cases were involved in the outbreak; however, clinical specimens could be collected from only two patients, both of whom were confirmed as acute CCHF cases using CCHF-specific reverse transcriptase polymerase chain reaction (RT-PCR). Phylogenetic analysis of the complete S, M, and L segment sequences places the Abyei strain of CCHF virus in Group III, a virus group containing strains from various countries across Africa, including Sudan, South Africa, Mauritania, and Nigeria. The Abyei strain detected in 2009 is genetically distinct from the recently described 2008 Sudanese CCHF virus strains (Al-fulah 3 and 4), and the Abyei strain S and L segments closely match those of CCHF virus strain ArD39554 from Mauritania.

**Conclusions:**

The present investigation illustrates that multiple CCHF virus lineages are circulating in the Kordufan region of Sudan and are associated with recent outbreaks of the disease occurring during 2008–2009.

## Author Summary

The tick-borne virus which causes the disease Crimean-Congo hemorrhagic fever (CCHF) is known to be widely distributed throughout much of Africa, Southern Europe, the Middle East, Central Asia, and Southern Russia. Humans contract the virus from contact with infected people, infected animals (which do not show symptoms), and from the bite of infected ticks. CCHF was recently recognized in the Sudan when several hospital staff and patients died from the disease in a rural hospital. The genetic analysis of viruses associated with the 2008 and 2009 outbreaks shows that several CCHF viral strains currently circulate and cause human outbreaks in the Sudan, highlighting CCHF virus as an emerging pathogen. The Sudanese strains are similar to others circulating in Africa, indicating movement of virus over large distances with introduction and disease outbreaks in rural areas possible. Understanding the epidemiology of zoonotic diseases such as CCHF is especially important in the Sudan given the large numbers of livestock in the country, and their importance to the economy and rural communities. It is imperative that hospital staff consider CCHF as a possible disease agent, since they are at a high risk of contracting the disease, especially in hospitals with limited medical supplies.

## Introduction

Crimean-Congo hemorrhagic fever (CCHF), caused by Crimean-Congo hemorrhagic fever virus (CCHFV), is a tick-borne virus with a tripartite RNA genome (segments designated S, M, and L). CCHFV can be transmitted to humans by the bite of an Ixodid tick and by contact with blood or tissue from infected livestock and humans [1]–[3].

CCHFV has been detected in a broad geographic zone including much of Africa, southern Europe and Asia, extending from western China to the Middle East and Southern Russia, where focal endemic areas have been identified [1], [4]–[13]. Disease outbreaks and sporadic cases of CCHF have been recorded throughout these endemic areas, with several recent outbreaks occurring in the Sudan [14]. In 2008, a nosocomial outbreak of CCHF occurred in a rural hospital in the Al-fulah District, Western Kordufan, Sudan [14]; two minor genetic variants, designated Al-fulah 3 and 4 (Genbank Q862371-2), were identified during that outbreak and were thought to be responsible for the emergence of the disease in the region.

We conducted the present study to analyze a June 2009 disease cluster involving approximately seven suspect cases of CCHF in Dunkop village, Abyei District, South Kordufan, Sudan. This represents the second CCHF outbreak reported in Sudan's Kordufan region last year. Although clinical samples could only be collected for two of the cases involved in the June outbreak, both cases were laboratory confirmed as acute CCHF by reverse transcription polymerase chain reaction (RT-PCR) targeting the viral nucleoprotein (NP) of the S segment, which is one of the least variable areas of the genome [15]. Genetic analysis of virus from one case-patient demonstrated that the virus strain belonged to Group III – a virus lineage associated with strains found in Sudan, South Africa, Mauritania, and Nigeria. However, additional analysis revealed that the virus strain implicated in the June 2009 cluster was genetically distinct from that involved in the 2008 Al-fulah outbreak.

## Materials and Methods

### Hemorrhagic Fever Case Definition

The case definition used for identification of suspect hemorrhagic fever patients included onset of fever and one or more of the following symptoms: hemorrhagic manifestations (ecchymosis, petechia, epistaxis, hematemesis), bloody diarrhea, severe headache, joint pain, chills, headache, and nausea.

### Sample Collection and Preparation

Blood samples were collected from two acute hemorrhagic fever (HF) patients in clean, sterile vacutainers. Samples were collected as part of routine diagnostic testing during outbreak response. Ethical clearance was obtained from the Ministry of Health of Sudan and informed consent from all patients was provided through an ethical clearance form which permitted use of the samples for diagnostic and research purposes. In addition to being used for routine malaria screening, blood was allowed to clot, and sera were separated and sent to the Division of Virology, National Medical Health Laboratory, Khartoum, Sudan, for diagnostic screening. Aliquots of serum samples were shipped frozen in Trizol buffer at a ratio of 1∶5 to the Viral Special Pathogens Branch, U.S. Centers for Disease Control and Prevention, Atlanta, GA for viral RNA extraction and subsequent sequencing of the whole virus genome.

### Virus Isolation and Serology

Due to the hazards associated with cultivation of CCHFV (the presumptive cause of disease), virus isolation attempts were not conducted in Sudan, and regulations prevented shipment of the infectious clinical samples to CDC's high-containment facility in Atlanta. Therefore, identification of the virus was solely dependent on serology and RT-PCR amplification, using primers targeting the S segment of the Al-fulah strain of CCHFV (Genbank accession number GQ862371), followed by direct sequencing.

Serologic tests were conducted in Sudan to screen the sera for antibodies to yellow fever and dengue fever using kits provided by NAMRU3, Cairo, Egypt. Rift Valley fever and CCHF virus antibody testing in Sudan was done using ELISA kits from Biological Diagnostic Supplies Limited (South Africa). Screening was performed using either IgM enzyme-linked immunosorbent assays or indirect immunofluorescence assays (IIFT).

### Molecular Diagnostic Testing

Viral RNA was extracted from the patient's serum samples either using QIAamp viral RNA kit (QIAamp, GmHb, Germany) or Qiagen's RNeasy kit as per manufacturer's instructions. The nucleoprotein gene was targeted, using a pair of primers designed based on alignments of nucleotide sequences of Al-fulah strain (Genbank accession number GQ862371) with several CCHFV strains using BioEidit software (Carlsbad, CA, USA). The forward primer CCHF1 (5′-ATA CGA GTG TGC ATG GGT CA-3′) included bases 286–305 of the positive sense strand of the virus S segment, whereas the reverse primer CCHF2 (5′-TTT GCA ATG TGC TTG AGG AG -3′) included bases 892-912 of the negative sense strand. Use of primers CCHF1 and CCHF2 in an RT-PCR produced a 627-bp primary PCR product. All primers were synthesized on a DNA synthesizer (Milliigen/Biosearch, a division of MilliporeBurlington, MA, USA) and purified using oligo-pak oligonucleotide purification columns (Glen Research Corporation, Sterling, VA, USA.) as per manufacturer's instructions.

Five microliters of extracted RNAs were employed in RT-PCR assays to detect the CCHF viral genome using primers either described in this study or by Schwartz et al [16]. The RNA specimens were also submitted to the Molecular Biology Laboratory of the Faculty of Veterinary Medicine, University of Khartoum, and the Faculty of Medical Laboratory Sciences, the National Ribat University, Sudan, for further confirmation of the results by RT-PCR as described by Schwartz et al [16]. Additional suspected hemorrhagic fever viruses including Rift Valley fever and yellow fever were ruled out by RT-PCR using previously described assays [17]. Detection of dengue virus serotypes was carried out using Geno-Sen's DENGUE 1–4 Real Time PCR Kit following the manufacturer's instructions (Genome Diagnostic Pvt. Ltd, India).

### Virus Complete Genome Sequencing

Sequences of the virus S, M, and L genome RNA segments were generated as previously described [15]. Viral RNA extracted from serum was used for RT-PCR amplification of all three segments for one patient from the Abyei outbreak (Genbank HQ378179, HQ378183, HQ378187). Additionally, sequences of two complete and one partial M segments (Genbank HQ378184-6) and three complete L segments (Genbank HQ378180-2) were generated from viruses detected in patient sera obtained from the previously reported Sudanese Al-fulah outbreak in 2008 [14].

### Phylogenetic Analysis

SeqView was used to align complete S, M, and L segment sequences for all CCHFV strains available in GenBank, as well as the sequences obtained from Abyei 1–2009 and Al-fulah 1, 2, 3, 4, 6, 7, 8, and 9–2008 virus strains. Maximum likelihood analysis was conducted for each segment using default settings in GARLI (v0.96b8)[18]. Bootstrap support values were created from 1,000 replicates, and 50% majority rule trees were generated for each of the segments. CCHFV strains are represented by their country of origin, strain name, and date of collection when available.

## Results

### Seven patients suspected of HF illness near Abyei District, Sudan

**Figure 1:**
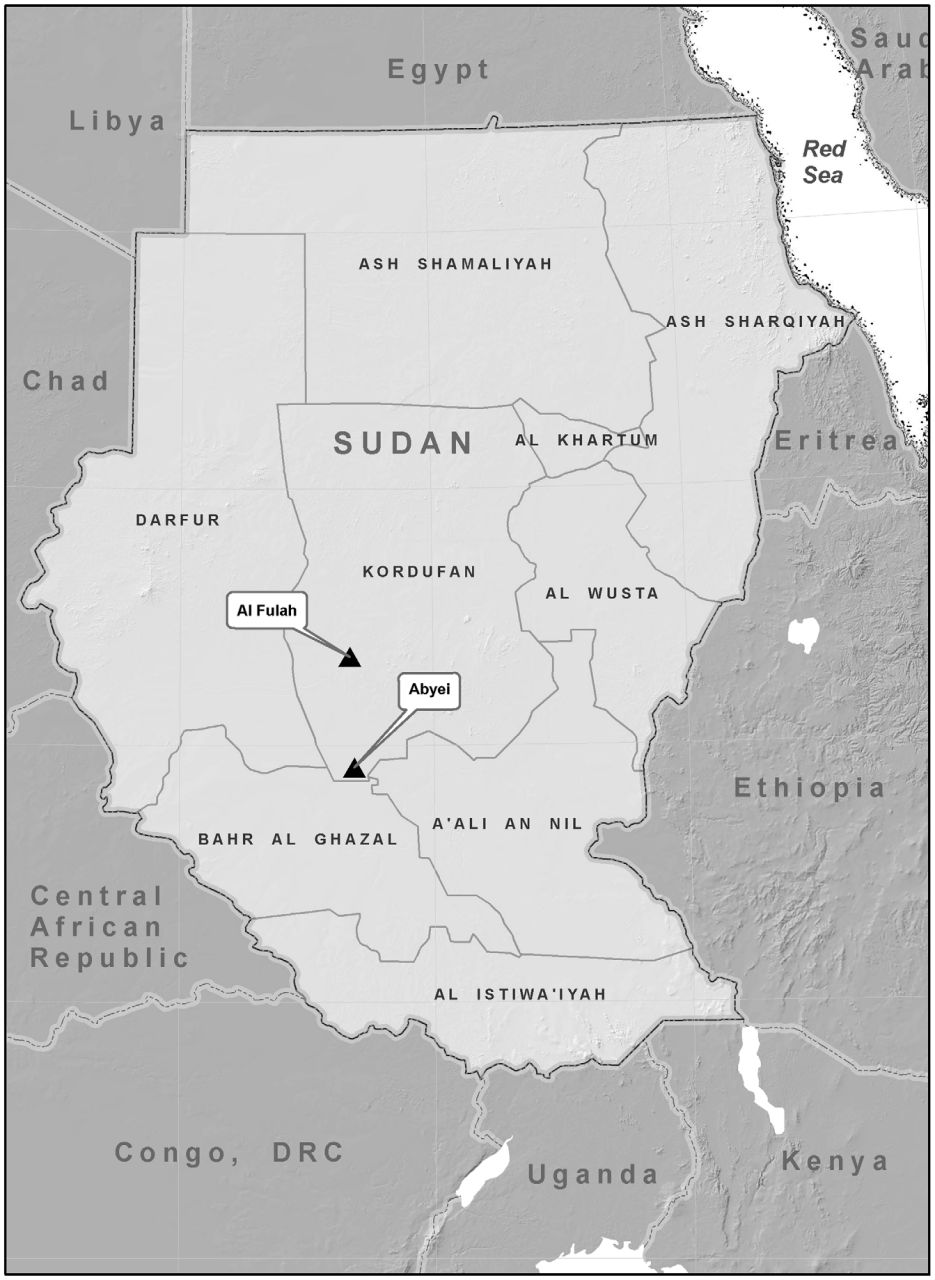
Map of Sudan showing the location of Abyei and Al-fulah towns in Kordufan region, Sudan.

During June 2009, seven patients meeting the hemorrhagic fever (HF) case definition were identified from Dunkop village. Dunkop village is located 15 kilometers to the east of Abyei in the former state of Western Kordufan, Sudan, an area that is shared in part by the government of South Sudan and the government of National Unity, Sudan. The population in Abyei District includes the nomadic Arab tribes of Misariya from the north and the Nilotic tribes of Dinka from the south. Transport from Dunkop village to the hospital in Abyei town is extremely difficult, if not impossible, during the rainy season, which includes the month of June. When passable, the distance between the primary-care centers and the surrounding villages is usually crossed by walking; therefore, evacuation of suspect cases from Dunkop village to Abyei hospital was not possible for all patients due to poor road conditions and prevailing insecurity at the time. Of the five patients able to travel to Abyei hospital, two died and one was discharged before samples could be collected for laboratory testing. Given the proximity of approximately 250 kilometers from Al-fulah (Figure 1), the location of a nosocomial outbreak of CCHF in October 2008, CCHF was considered as a possible cause of the HF illness [14]. Blood samples were available from two patients with signs of acute HF illness. The extracted RNA samples were used in RT-PCR as targets for CCHF definitive diagnosis and subsequent sequencing.

Limited epidemiologic and clinical details were obtained where possible from interviews with patients or close relatives, or from hospital records (Table 1). The age range of the seven patients suspected of having CCHF was 21–65 years. Five of the suspect patients were female (all housewives), and the last two were male (a student and a day laborer). Traditionally, the activities of women in these rural villages include shepherding livestock and milking of cows, and both the index case and one of the confirmed cases specifically reported animal contact. The potential for exposure to viremic livestock or virus infected ticks would be considered high. No evidence of nosocomial transmission was apparent based on the lack of any reports of infections with compatible illness among health care workers potentially exposed to these cases. All cases were from the same small village, and although there were reports of contact between several of the cases, no strong evidence of a clear chain of transmission could be discerned based on the limited data available. Contact transmission cannot be ruled out however, as the dates for onset of symptoms ranged over a 6 day period from June 12^th^ through 18^th^ 2009, allowing for the possibility of later cases having acquired the infection from the earliest cases identified.

**Table 1:** Suspect hemorrhagic fever case characteristics.

Patient Number	Age	Occupation	Sex	CCHF RT-PCR	Date Symptom Onset	Date of Death	Symptoms
1	40	housewife	F	ns	6/13/2009	6/15/2009	fever, headache, nausea, agitation, vomiting blood
2	55	housewife	F	ns	6/12/2009	6/15/2009	fever, diarrhea, bleeding from nostrils
3	44	housewife	F	ns	6/14/2009	survived	fever, diarrhea, bleeding from nostrils
4	30	housewife	F	ns	6/14/2009	survived	fever, diarrhea, bleeding from nostrils
5	65	housewife	F	ns	6/15/2009	6/20/2009	fever, headache, swollen joints, chest pain, agitation, dysentery, vomiting blood, bleeding from nostrils, GI bleeding, bleeding from mouth, coma
6	21	student	M	Positive	6/15/2009	6/21/2009	fever, headache, nausea, agitation, vomiting blood
7	35	day laborer	M	Positive	6/18/2009	survived	fever, headache, nausea, vomiting, abdominal pain, joint pain, epistaxis, conjunctivitis

ns = no sample.

Symptoms and clinical signs of the disease among the seven suspected patients, included rapid onset of fever (common to all suspected patients), and some or all of the following symptoms: headache; nausea; vomiting (with or without blood); severe muscle, joint, and/or chest pain; agitation; diarrhea; bleeding from the nostrils, mouth, gum, and upper and lower gastrointestinal tracts. Four of the seven cases progressed to coma and fatal outcome.

### Two case-patients tested positive for CCHF by RT-PCR

Serologic tests on sera from two suspected HF patients were negative for yellow fever, dengue fever, West Nile fever, and Rift Valley fever, as determined by either IgM enzyme-linked immunosorbent assay (ELISA) or indirect immunofluorescence assay (IIFT). Sera from these patients also tested negative for the presence of CCHF antibodies, as is common in fatal CCHF cases when sera is drawn 1–3 days post onset of illness. A second blood sample was not available from the surviving patient for further serological testing despite having been hospitalized for 2 days before discharge.

RT-PCR assays of RNAs extracted from sera of suspected HF patients were negative for Rift Valley fever, yellow fever, and dengue viruses; however, conventional RT-PCR resulted in amplification of a 627-bp PCR product specific for the CCHFV S segment (data not shown). The two RNA specimens were submitted to the Molecular Biology Laboratories at the University of Khartoum and the National Ribat University, and were confirmed as CCHF cases by RT-PCR using the same primers described in this study and CCHF primers described by Schwartz et al [16] (data not shown). In addition, blood smears were obtained for malaria testing at Abyei hospital, and both were positive. The finding of positive malaria smears is not uncommon in this area of rural Sudan and the clinical significance of the finding in these cases was difficult to evaluate.

### CCHF virus strain detected in Abyei in 2009 belongs to Group III

Using the method reported earlier [15], whole CCHF virus genome (S, M and L RNA segments) sequencing was attempted for each of the CCHF virus PCR positive specimens collected from the June 2009 Abyei outbreak and those from the earlier Al-fulah CCHF outbreak [14]. The complete sequence of the virus S, M and L genomic segments was successfully obtained for one sample from the Abyei outbreak, and two complete and one partial M segments, and two full L segments were generated from the earlier Al-fulah specimens (Table 2).

**Table 2:** Genbank accession numbers for Sudanese CCHF strains.

Sample Name	S	M	L
Al-fulah 1	Full GQ862371		
Al-fulah 2	Full GQ862371		
Al-fulah 3 (reference)	Full GQ862371	Full HQ378184	Full HQ378180
Al-fulah 4	Full GQ862372	Partial (∼4 kb) HQ378186	Full HQ378181
Al-fulah 6	Full GQ862371		
Al-fulah 7	Full GQ862371		
Al-fulah 8	Full GQ862371		
Al-fulah 9	Full GQ862371	Full HQ378185	Full HQ378182
Abyei 1 (reference)	Full HQ378179	Full HQ378187	Full HQ378183

Comparison of the virus sequences detected in the 2008 Al-fulah outbreak revealed that few mutations occurred in any of the three segments. Only one nucleotide difference was found among the virus S segments; both the partial Al-fulah 4-2008 and complete Al-fulah 9-2008 M segment sequences had one base change when compared to the reference Al-fulah 3–2008 sequence. The complete L segment sequences for viruses Al-fulah 4–2008 and 9–2008 contained two and one nucleotide differences, respectively, when compared to Al-fulah 3–2008. These results indicated circulation of identical or near-identical virus variants within the outbreak, consistent with the report of most of these cases being linked by nosocomial transmission [14].

Phylogenetic analysis of the virus sequences derived from the Abyei outbreak samples demonstrated that this outbreak was also caused by a virus belonging to Group III [15].

### Multiple strains of CCHF have been circulating in Sudan in recent years

**Figure 2:**
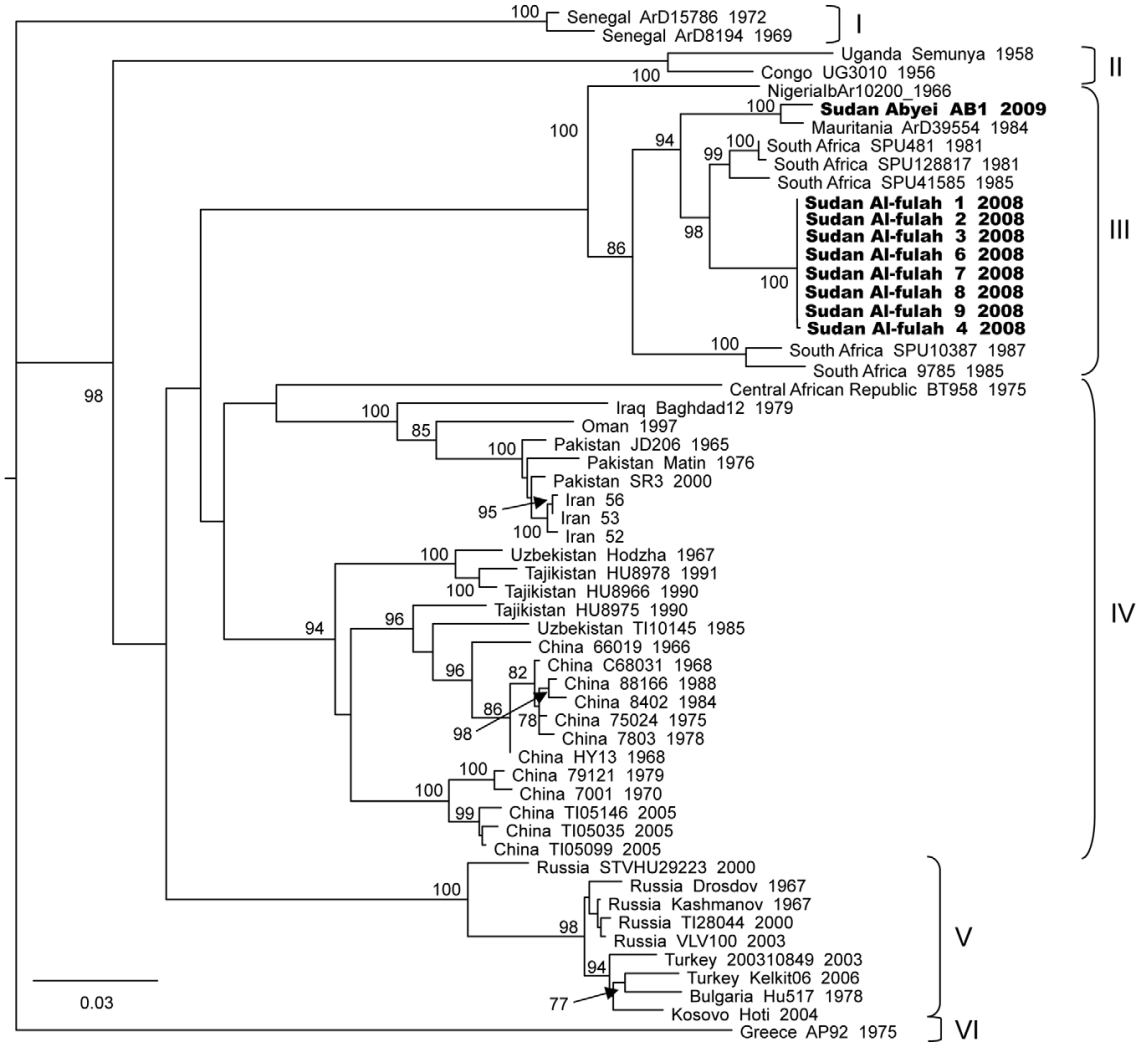
Phylogenetic relationship of Crimean-Congo hemorrhagic fever virus full length S segments. Phylogenetic relationship of the S segment sequence of the CCHF virus Abyei and Al-fulah strains relative to previously published sequences was carried out using GARLI (v0.96b8) [18], with default settings to generate a maximum likelihood tree with bootstrap support values from 1000 replicates. The 50% majority rule tree is depicted. Each strain is listed by its location, strain name, and year of isolation when available with the Sudanese strains bolded.

**Figure 3:**
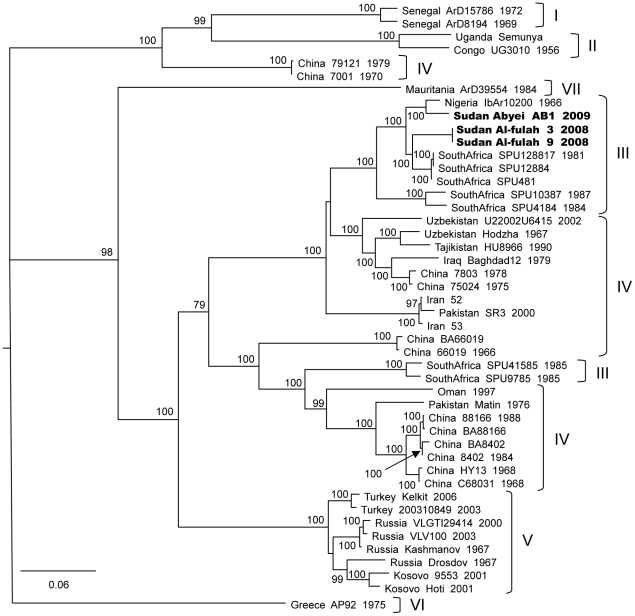
Phylogenetic relationship of Crimean-Congo hemorrhagic fever virus full length M segments. Phylogenetic relationship of the M segment sequence of the CCHF virus Abyei and Al-fulah strains relative to previously published sequences was carried out using GARLI (v0.96b8) [18], with default settings to generate a maximum likelihood tree with bootstrap support values from 1000 replicates. The 50% majority rule tree is depicted. Each strain is listed by its location, strain name, and year of isolation when available with the Sudanese strains bolded.

**Figure 4:**
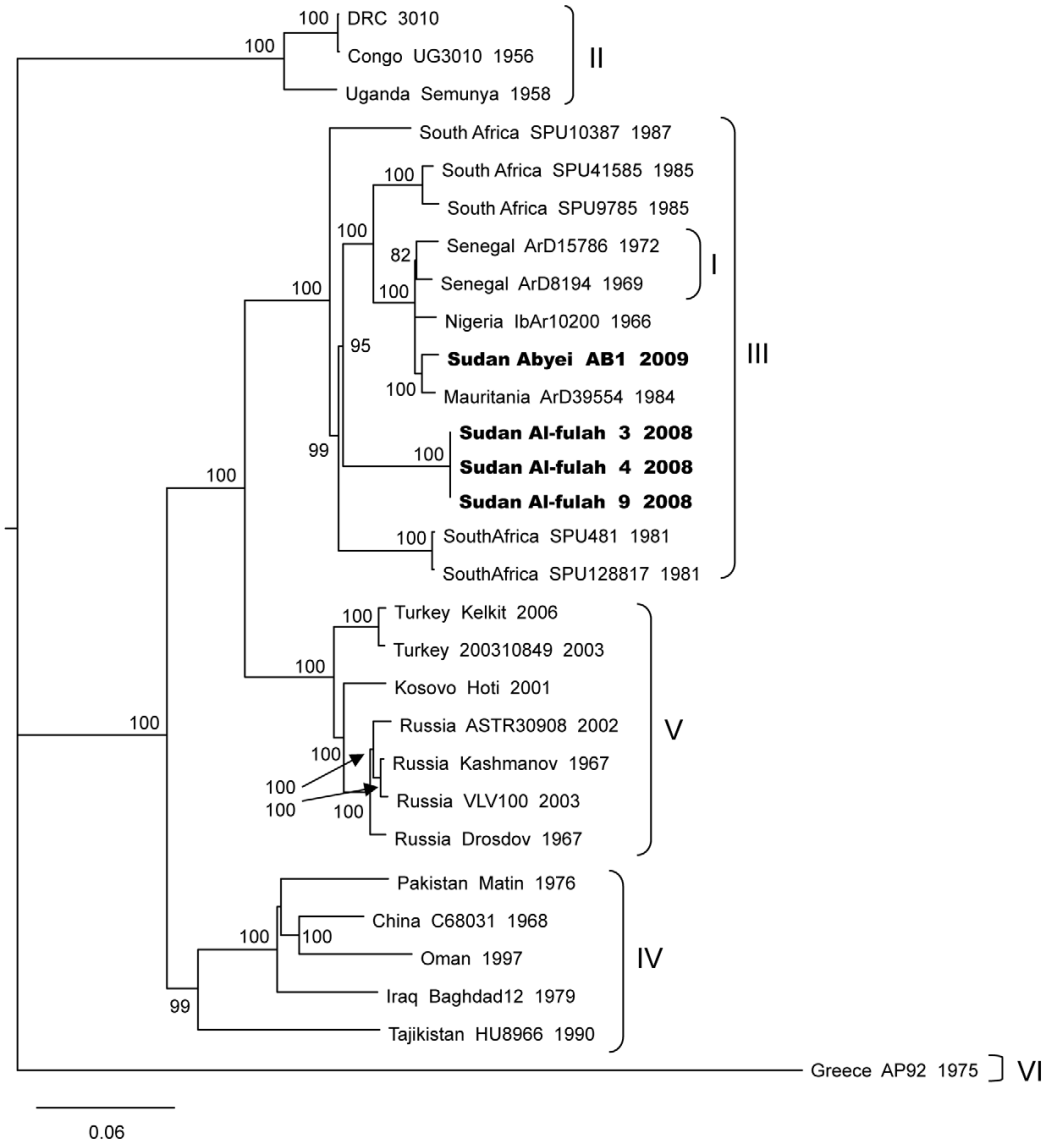
Phylogenetic relationship of Crimean-Congo hemorrhagic fever virus full length L segments. Phylogenetic relationship of the L segment sequence of the CCHF virus Abyei and Al-fulah strains relative to previously published sequences was carried out using GARLI (v0.96b8) [18], with default settings to generate a maximum likelihood tree with bootstrap support values from 1000 replicates. The 50% majority rule tree is depicted. Each strain is listed by its location, strain name, and year of isolation when available with the Sudanese strains bolded.

While viruses from the Al-fulah 2008 and Abyei 2009 outbreaks are both located in Group III, the Abyei strain was genetically distinct from the Al-fulah strain (Figures 2, 3, and 4). The two Sudanese strains both displayed closer genetic relationships with other Group III viruses from elsewhere in Africa than with one another. The S and L segments of the Abyei strain matched most closely with those of the Group III Mauritanian virus strain ArD39554 (Figures 2 & 4). This virus had been shown previously to be an M segment reassortant virus with the virus M segment being the sole representative of a highly unique Group VII [15], [19]. The M segment of the Abyei strain matched another Group III virus, namely the Nigerian IbAr10200 strain, consistent with the Abyei virus not being a reassortant (Figure 3). The Al-fulah strain genome segments matched most closely to other Group III viruses from South Africa. No evidence of reassortment or recombination was found for either of the Sudanese CCHFV strains [9], [13], [20]–[21].

## Discussion

In Sudan, sero-epidemiological surveys conducted over the past few decades have suggested the presence of various arboviruses in human populations [22]–[23]. In addition, CCHFV specific antibodies have been detected in camels in Egypt, and in sheep and goats in Jeddah, Saudi Arabia, all of which had been imported to these countries from Sudan [24]–[25].

Recently, CCHFV activities have been reported in the Kordufan region of Sudan. Several suspected sporadic cases and small outbreaks of CCHF associated with high case-fatalities were reported in rural hospitals from October 2008 through June 2009. CCHF was first confirmed to be present in Sudan during the 2008 nosocomial outbreak in a rural hospital in Al-fulah, Western Kordufan [14]. During the Al-fulah outbreak, the initial treatment in the hospital focused primarily on malaria, as blood smears from some of the CCHF suspect patients were positive for malaria. Both Plasmodium falciparum and P. vivax are found in Sudan, and malaria is endemic in Kordufan region, with the transmission season lasting from June/July to October/November [26]–[27]. Given how common positive smears are in this locale, it is difficult to assess the clinical significance of the positive smears reported in these two lab confirmed CCHF cases, particularly in the absence of identification of Plasmodium species involved. While speculative, it is possible that the immunosuppression associated with CCHFV infections [28] might facilitate malaria recurrence and invasion of malaria parasites from the liver to the blood stream. Our point is that the finding of a malaria positive smear should not be basis of cessation of testing for other etiologies which pose a risk of nosocomial transmission to the medical staff, as fever of unknown origin can also indicate viral hemorrhagic fever (VHF) in endemic countries like Sudan [14]. In this outbreak, serum samples from the patients were also tested for CCHF which led to the identification of etiology of the disease outbreak.

Transmission of CCHFV in the recent Sudanese outbreaks likely involved either tick-bites or person-to-person spread. Tick bite transmission or exposure to blood or tissues of infected livestock is more likely to occur during rainy months, when roads become very muddy and transport between villages or cities, becomes extremely difficult. In these conditions, the local residents typically live in close contact with their cattle within their home villages, increasing the risk of acquiring infection from infected livestock or by tick bite. Once CCHF cases are admitted to medical facilities, nosocomial chains of transmission can occur within hospitals and clinics lacking resources or training to fully implement universal barrier nursing conditions and effective infection control practices [29]–[30]. This was clearly the case in the Al-fulah 2008 outbreak [14].

The genetic diversity of CCHFV, its virulence, and its potential as a bioterrorism agent underscore the importance of genetic characterization of virus strains from all geographically distinct endemic areas. This type of analysis improves the design and development of rapid diagnostics and potential candidate vaccines for this devastating disease.

This investigation expands on existing data indicating that Group III CCHFV lineage, known to be endemic in Sudan, South Africa, Mauritania, and Nigeria, is broadly distributed within Africa. Furthermore, our study illustrates that multiple strains belonging to Group III virus lineage from the African continent are currently present in the Sudan and are responsible for the recent outbreaks of the disease in the Kordufan region. The genetic linkage of the recent Sudan outbreak viruses would be compatible with viruses having been introduced to Sudan from South Africa, Senegal, Mauritania or Nigeria as a result of the international trade of livestock or by bird migration. However, as the Sudan is the largest country in Africa and the richest in animal resources, it seems unlikely to import livestock and associated products from these distant regions of Africa. Phylogenetic study of the sequence diversity of all the characterized CCHFV strains clearly indicates that virus genetic lineages are not precisely restricted to defined geographic locations, but considerable movement of viruses is occurring over long distances. These data would suggest that the role of bird migration in disease transmission should strongly be considered in the dissemination of CCHFV.

The occurrence of multiple CCHF outbreaks in Sudan in the past few years, and the risks these cases pose for medical staff in resource poor health care facilities indicate the importance of improved surveillance in Sudan for this important disease. The attending physicians, working in those areas of endemicity, should consider this virus in their efforts to diagnose the disease in patients presented to hospitals or clinical centers with symptoms indicative of HF.
